# Uncovering the Causal Link Between Obesity‐Associated Genes and Multiple Sclerosis: A Systematic Literature Review

**DOI:** 10.1002/brb3.70439

**Published:** 2025-04-07

**Authors:** Ali Jafari, Sara Khoshdooz, Melika Arab Bafrani, Farnush Bakhshimoghaddam, Hamid Abbasi, Saeid Doaei

**Affiliations:** ^1^ Student Research Committee, Department of Community Nutrition, Faculty of Nutrition Sciences and Food Technology, National Nutrition and Food Technology Research Institute Shahid Beheshti University of Medical Sciences Tehran Iran; ^2^ Systematic Review and Meta‐analysis Expert Group (SRMEG) Universal Scientific Education and Research Network (USERN) Tehran Iran; ^3^ Faculty of Medicine Guilan University of Medical Science Rasht Iran; ^4^ Faculty of Medicine Tehran University of Medical Sciences Tehran Iran; ^5^ Department of Nutrition, School of Allied Medical Sciences Ahvaz Jundishapur University of Medical Sciences Ahvaz Iran; ^6^ Student Research Committee Tabriz University of Medical Sciences Tabriz Iran; ^7^ Neurosciences Research Center Tabriz University of Medical Sciences Tabriz Iran; ^8^ Department of Community Nutrition, Faculty of Nutrition and Food Technology, National Nutrition and Food Technology Research Institute Shahid Beheshti University of Medical Sciences Tehran Iran

**Keywords:** gene, multiple sclerosis, obesity

## Abstract

**Background:**

Multiple sclerosis (MS) is a multifaceted neurodegenerative disorder influenced by genetics and lifestyle. This systematic literature review investigates the role of six obesity‐associated genes, including fat mass and obesity‐associated (*FTO*), FAS apoptosis inhibitory molecule 2 (*FAIM2*), Niemann–Pick disease type C1‐like 1 (*NPC1*), glucosamine‐6‐phosphate deaminase 2 (*GNPDA2*), melanocortin‐4 receptor (*MC4R*), and brain‐derived neurotrophic factor (*BDNF*) in the context of MS.

**Methods:**

A literature search was executed using Embase, Scopus, Cochrane, Web of Science, and PubMed databases from inception to July 2024. The related keywords employed during the search process are “fas apoptotic inhibitory molecule 2,” “Niemann–Pick disease type C1,” “fat mass and obesity‐associated,” “melanocortin‐4 receptor,” “brain‐derived neurotrophic factor,” “glucosamine‐6‐phosphate deaminase 2,” and “multiple sclerosis.”

**Results:**

Out of 2108 papers, 27 were entered into the present systematic literature review. The *FTO* gene may affect MS susceptibility through metabolic and inflammatory pathways. *FAIM2* and *NPC1* genes may contribute to MS pathogenesis, though their precise roles are still being elucidated. The *GNPDA2* gene may have some connections with MS but requires further clarification. *MC4R* has demonstrated significant neuroprotective and anti‐inflammatory effects, suggesting its potential impact on MS progression. *BDNF* plays a complex role in neuronal survival and repair and may influence the risk of MS.

**Conclusion:**

Our findings demonstrated that obesity‐related genes may have a significant impact on MS risk and disease course, revealing novel insights into the genetic underpinnings of MS.

## Graphical abstract

This systematic literature review explores the influence of six obesity‐associated genes on multiple sclerosis (MS) risk and progression, revealing significant genetic underpinnings and potential pathways for MS pathogenesis and treatment.

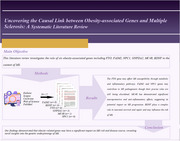

**Abbreviations**
AICD: activation‐induced cell death
ACTH: adrenocorticotropic hormone
BBB: blood‐brain barrier
BMI: body mass index
BDNF: brain‐derived neurotrophic factor
CNTF: ciliary neurotrophic factor
CIS: clinically isolated syndrome
cAMP‐PKA‐CREB: cyclic AMP‐protein kinase A‐cAMP response element‐binding
EAE: experimental autoimmune encephalomyelitis
FAIM2: fas apoptotic inhibitory molecule 2
FTO: fat mass and obesity‐associated
FGF: fibroblast growth factor
FGFRs: fibroblast growth factor receptors
Fru6P: fructose‐6‐phosphate
GWAS: genome‐wide association studies
GA: glatiramer acetate
GlcN6P: glucosamine‐6‐phosphate
GNPDA2: glucosamine‐6‐phosphate deaminase 2
GFAT: glutamine‐fructose‐6‐phosphate aminotransferase
HBP: hexosamine biosynthesis pathway
IMSGC: International MS Genetics Consortium
IFN‐β: interferon‐beta
DEGs: key differentially expressed genes
LFG: lifespan‐enhancing gene
MC4R: melanocortin‐4 receptor
miRNAs: MicroRNAs
NPC1: Niemann–Pick disease type C1
NGF: nerve growth factor
NT‐3: neurotrophin‐3
PPMS: primary‐progressive MS
ROS: reactive oxygen species
RRMS: relapsing‐remitting MS
SPMS: secondary‐progressive MS
SNPs: single nucleotide polymorphisms
TNF‐α: tumor necrosis factor‐alpha
α‐MSH: α‐melanocyte‐stimulating hormone

## 1 Introduction

Multiple sclerosis (MS) is a chronic neuroimmune‐mediated disease characterized by central nervous system (CNS) demyelination, affecting over 2.9 million individuals globally (Lubetzki and Stankoff 2014; Walton et al. 2020). The average age of MS onset typically ranges from 20 to 40 years; however, many cases have been reported in both older and younger populations (Hansen and Okuda 2018; Reinhardt et al. 2014). MS manifests in several subtypes, including relapsing–remitting MS (RRMS), secondary‐progressive MS (SPMS), primary‐progressive MS (PPMS), and clinically isolated syndrome (CIS) (Lublin et al. 2014; Thompson et al. 2018). The clinical presentation of MS is diverse, encompassing sensory and motor deficits, fatigue, cognitive decline, psychiatric symptoms, optic neuritis, and movement disorders (Javalkar et al. 2016). While the precise etiology of MS remains elusive, both genetic and environmental factors are recognized as critical contributors to MS development (Axisa and Hafler 2016; Jörg et al. 2016). A multifaceted approach is typically employed in managing patients with MS (Hauser and Cree 2020). Recent evidence underscores the significant impact of maintaining a healthy lifestyle and following a balanced diet on the progression and pathogenesis of MS (Mentis et al. 2021; Snetselaar et al. 2023; Stoiloudis et al. 2022; Wahls 2022), with low vitamin D levels and obesity identified as notable risk factors (Gombash et al. 2022; Lutfullin et al. 2023).

Obesity, characterized by excessive fat accumulation, is increasingly recognized as a risk factor for a range of neurological diseases, including Parkinson's disease, dementia‐related disorders, amyotrophic lateral sclerosis, and MS (Dardiotis et al. 2018; Giannopapas et al. 2024; Rahmani et al. 2022; Schwartz et al. 2017; Su et al. 2021). A recent meta‐analysis of individuals with MS reported a mean body mass index (BMI) of 25.73 kg/m^2^ (Dardiotis et al. 2019). Moreover, studies in both adults and children demonstrated a 2‐fold increase in MS risk among obese individuals (BMI > 30 kg/m^2^) compared to those with a BMI of 18.5–21 kg/m^2^ (Gianfrancesco et al. 2017). The low‐grade neuroinflammation associated with obesity is thought to contribute to MS progression and disability (Castro et al. 2019; Stampanoni Bassi et al. 2020), with evidence suggesting a positive link between obesity and increased MS disability, potentially leading to an earlier transition from RRMS to SPMS (Fitzgerald et al. 2020; Manouchehrinia et al. 2018; Pilutti and Motl 2019). Additionally, pediatric obesity has been linked to both early (Langer‐Gould et al. 2013) and later (Munger et al. 2013) MS onset.

On the other hand, genome‐wide association studies (GWAS) uncovered that multiple genetic variants are significantly related to an increased risk of obesity (R. K. Singh et al. 2017), particularly in environments conducive to obesity (Mintziori et al. 2020; Thaker 2017). To date, over 300 gene polymorphisms have been documented (Zhao et al. 2014). Key obesity‐associated genes include fat mass and obesity‐associated (*FTO*), FAS apoptotic inhibitory molecule 2 (*FAIM2*), Niemann–Pick disease type C1 (*NPC1*), melanocortin‐4 receptor (*MC4R*), glucosamine‐6‐phosphate deaminase 2 (*GNPDA2*), and brain‐derived neurotrophic factor (*BDNF*) (Ang et al. 2023). Interestingly, recent research has identified that various genetic variants in the genes that are associated with obesity‐related traits may have a key role in the genetic predisposition of MS.

The purpose of this systematic literature review was to explore the link between obesity and MS, with a focus on obesity‐associated genes. This study also aimed to elucidate the possible mechanisms underlying the effects of obesity‐associated genes on the MS pathogenesis.

## 2 Methods

This systematic literature review was executed in accordance with the guidelines outlined by the Preferred Reporting Items for Systematic Reviews and Meta‐analyses (PRISMA) framework (Page et al. 2021).

### 2.1 Search Strategy

An advanced literature search was conducted using Embase, Scopus, Cochrane, Web of Science, and PubMed databases. The search included all relevant publications from inception to July 2024, employing the related keywords: “fas apoptotic inhibitory molecule 2,” “Niemann–Pick disease type C1,” “fat mass and obesity‐associated,” “melanocortin‐4 receptor,” “brain‐derived neurotrophic factor,” “glucosamine‐6‐phosphate deaminase 2,” and “multiple sclerosis” (Table S1). Additionally, the references of all selected papers were reviewed to ensure comprehensive coverage and to prevent the omission of relevant studies. The acquired data were extracted from human studies, comprising the authors’ first names, country, study design, sample size, gene polymorphism, and findings.

### 2.2 Eligibility Criteria and Selection Process

Studies investigating the relationship between obesity‐associated genes—including *FTO*, *FAIM2*, *NPC1*, *MC4R*, *GNPDA2*, and *BDNF*—and *MS* were selected. The selection process was independently carried out by four reviewers (M.A.B., F.B.M., A.J., and S.K.). In cases of disagreement between the reviewers, two additional authors (H.A. and S.D.) were consulted to resolve discrepancies through discussion. Studies deemed irrelevant to the focus of this systematic literature review were excluded. The PECO framework used in this systematic literature review encompassed the population (individuals with MS), exposure (obesity‐associated genes), comparison (with or without controls), and outcome (risk of MS).

### 2.3 Data Extraction

Two researchers (F.B.M. and H.A.) independently extracted the necessary data using a predesigned Microsoft Word table, which was then reviewed by the senior author (S.D.). The extracted data were obtained from human studies and included the authors’ first names, country, study design, sample size, gene polymorphism, and findings, as depicted in Table 1.

**TABLE 1:** Characteristics of selected human papers exploring the link between obesity‐associated genes and multiple sclerosis.

Study	Country	Study design	Sample size (case‐control)	Gene polymorphisms	Findings	Methodological quality assessment
Al‐Serri et al. (2019)	Kuwait	Cohort	140	*FTO* rs9939609	The A‐allele is associated with being overweight/obese and increased disability in MS patients, but not with the risk of developing MS.	High
Kamermans et al. (2019)	The Netherlands	Case‐control	11/6	*MC4R*	MC4R mRNA and protein are expressed on astrocytes, and increased astrocytic MC4R expression was observed in active MS lesions. The highly selective MC4R agonist setmelanotide ameliorated astrocyte's reactive phenotype in vitro and induced interleukin‐6 and ‐11 production, possibly through increased CREB phosphorylation.	High
Mokry et al. (2016)	UK	Mendelian randomization study	GIANT (*n* = 322,105); IMSGC (*n* = 14,498 cases and 24,091 controls)	70 SNPs	The findings indicate that an increased BMI influences susceptibility to MS, with a 1 standard deviation rise in genetically determined BMI (kg/m^2^) leading to a 41% increase in the odds of MS.	High
Davis et al. (2014)	South Africa	Case‐control	114/195	*FTO* rs9939609	The FTO rs9939609 A‐allele, associated with increased homocysteine levels in MS patients but not in controls, showed a positive correlation with BMI and TC levels.	High

Abbreviations: CREP, cAMP responding element binding protein; FAIM2, FAS apoptotic inhibitory molecule 2; FTO, fat mass and obesity‐associated; GIANT, Genetic Investigation of Anthropometric Traits; IMSGC, International MS Genetics Consortium; MC4R, melanocortin‐4 receptor; MS, multiple sclerosis.

### 2.4 Methodological Quality Assessment

The risk of bias in observational studies, such as case‐control and cohort studies, was assessed using the Newcastle–Ottawa Scale (NOS) by two independent researchers (H.A. and S.D.). This tool evaluates study quality across three key domains: selection (four criteria), comparability (one criterion), and exposure (three criteria), with a total possible score ranging from 0 to 9. Studies achieving a score of 7 or higher were classified as high‐quality (Table S2) (Stang 2010). The methodological quality of one Mendelian randomization (MR) study incorporated into the systematic literature review was assessed using an adapted version of the strengthening the reporting of observational studies in epidemiology for MR (STROBE‐MR) guidelines (Skrivankova et al. 2021). Quality scores were transformed into percentages; studies with scores below 75% were categorized as poor quality, those between 75% and 85% were classified as moderate quality, and studies exceeding 85% were designated as high quality (Table S3) (Boef et al. 2015; Davies et al. 2018). The final results of the methodological quality assessment of the selected studies are available in Table 1.

## 3 Results

### 3.1 Study Selection

**FIGURE 1:**
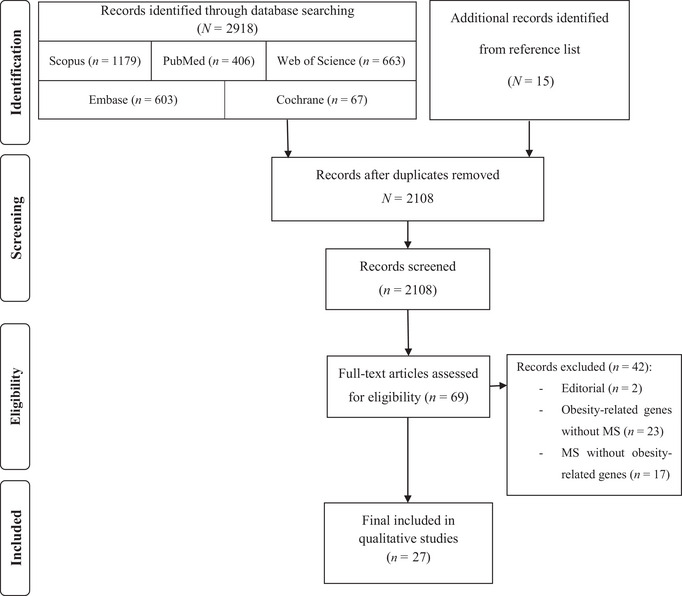
Systematic literature review flow chart.

A comprehensive evaluation was executed on 2108 attained papers, of which 27 were selected for inclusion in the present systematic literature review (Figure 1). Specifically, nine papers were identified concerning *FAIM2*, six pertaining to *FTO*, three associated with *GNPDA2*, one relevant to *MC4R*, and eight linked to *BDNF*. Notably, no study was identified for the *NPC1* gene in individuals with MS. Out of the 27 selected papers, only four were human studies, which included two case‐control studies, one cohort study, and one MR study, as presented in Table 1.

### 3.2 Methodological Quality Assessment

The risk of bias in the three selected observational studies was assessed using the NOS. Two selected studies scored 7 points, indicating high quality, with strong case definition, study representativeness, and reliable exposure and outcome measures (Al‐Serri et al. 2019; Davis et al. 2014). Another study scored 9 points and was also classified as high quality, with excellent study selection and exposure/outcome ascertainment, along with thorough confounder adjustment (Kamermans et al. 2019). Furthermore, for one MR study, all criteria were fully met, including the use of genome‐wide significant single nucleotide polymorphisms (SNPs), robust methods for assessing pleiotropy (MR‐Egger regression, weighted median), and sensitivity analyses to account for proxy SNPs and potential confounding factors. The study's high methodological rigor resulted in a high‐quality rating of 100% (Mokry et al. 2016). The final results of the methodological quality assessment of the selected studies are available in Table 1.

### 3.3 *FAIM2* Gene Polymorphisms and MS

*FAIM2*, also known as the lifespan‐enhancing gene (LFG), encodes a protein that plays a crucial role in inhibiting apoptosis, particularly in response to FAS receptor activation (Pawar et al. 2017). *FAIM2*'s antiapoptotic function is essential in various cell types, including neurons and immune cells, where FAIM2 promotes cell survival under stress conditions, thereby enhancing resilience against apoptotic signals (R. Singh et al. 2019).

*FAIM2* has been linked to obesity through associations with body fat distribution and BMI, two critical determinants of metabolic health (Corella et al. 2014). Specific SNPs in the *FAIM2* gene have been related to higher BMI and altered fat distribution, suggesting that *FAIM2* may influence key metabolic processes underlying obesity (Littleton et al. 2024). This connection is possibly relevant in the context of MS, where obesity is recognized as both a risk factor and a potential exacerbator of disease progression.

*FAIM2*'s role in immune regulation, especially within T cells, is of significant importance. *FAIM2* is expressed in various immune cells, including T lymphocytes, and modulates apoptotic pathways activated by FAS receptor signaling (Cai et al. 2022; Soliman et al. 2022). FAS‐mediated apoptosis is a vital mechanism for maintaining immune homeostasis by eliminating excess or autoreactive immune cells (Rossin et al. 2019). However, *FAIM2*‐induced inhibition of this process can promote the survival of immune cells, including potentially autoreactive T cells, contributing to the persistence and chronicity of the autoimmune response observed in MS (Xiang et al. 2023).

Apoptosis dysregulation is a key feature of MS, characterized by an imbalance between proapoptotic and antiapoptotic signals in immune cells. This imbalance permits the survival of autoreactive T cells that drive autoimmune‐mediated demyelination in MS, thereby perpetuating the disease (Kennedy et al. 2022). Normally, apoptosis helps eliminate these harmful cells in order to maintain immune tolerance, but in MS, this process is impaired, contributing to ongoing autoimmunity (Kennedy et al. 2022; Pukoli and Vécsei 2023).

Through its antiapoptotic function, *FAIM2* plays a significant role in apoptosis dysregulation by inhibiting Fas receptor‐mediated apoptosis of autoreactive T cells. By preventing apoptosis, *FAIM2* may contribute to the continued survival of autoreactive T cells and exacerbate the disease process in MS (Cai et al. 2022). This effect is notably pronounced in the context of obesity, which is often associated with chronic low‐grade systemic inflammation. Obesity‐induced inflammation is characterized by elevated levels of pro‐inflammatory cytokines, such as tumor necrosis factor‐alpha (TNF‐α) and IL‐6, which can further modulate *FAIM2* expression and activity in immune cells (Kawai et al. 2021; Zatterale et al. 2020).

The interaction between obesity and *FAIM2*‐mediated apoptosis inhibition fosters a sustained inflammatory environment that not only promotes the survival of autoreactive T cells but also enhances their activity (Hildebrandt et al. 2023). This prolonged inflammatory state, driven by both metabolic disturbances linked to obesity and impaired apoptotic mechanisms influenced by *FAIM2*, may amplify autoimmune attacks on myelin, resulting in more severe neuroinflammation and neurodegeneration (Ramírez‐Carreto et al. 2023). Consequently, the convergence of obesity‐related inflammation and *FAIM2*'s antiapoptotic effects could significantly worsen the clinical trajectory of MS, underscoring the need for further research into targeted therapeutic strategies that address this complex interplay (Correale and Marrodan 2022; Schreiner and Genes 2021; Stampanoni Bassi et al. 2020). These cytokines perpetuate immune‐mediated damage to myelin and exert direct cytotoxic effects on neurons and glial cells. Additionally, the high levels of oxidative stress in MS, stemming from an imbalance between reactive oxygen species (ROS) production and antioxidant defenses, further compromise neuronal integrity (Ohl et al. 2016; Olufunmilayo et al. 2023).

The interplay between *FAIM2*, obesity, and MS underscores the disease's complexity and highlights the need for a deeper understanding of the underlying molecular mechanisms. Elucidating the specific pathways through which *FAIM2* influences inflammatory and apoptotic processes in obese MS patients could reveal novel therapeutic targets. Such insights could pave the way for developing interventions that modulate *FAIM2* activity to reduce the inflammatory burden and slow MS progression in individuals with comorbid obesity.

Some studies indicated that specific polymorphisms in the *FAIM2* gene might be associated with an increased risk of developing MS, particularly among obese individuals (Kang et al. 2020). FAIM2 may influence metabolic pathways such as those involving insulin resistance and dyslipidemia, thereby modifying the overall disease trajectory in MS patients. Specifically, *FAIM2* might affect the activity of adipokines—signaling molecules secreted by adipose tissue—that play roles in both metabolic processes and MS pathology. By modulating the levels and activity of these adipokines, *FAIM2* could impact systemic inflammation and metabolic disturbances, further influencing disease outcomes in the context of obesity (Cho et al. 2023; Correale and Marrodan 2022; Park and Shimokawa 2024). These potential pathways highlight the need for further research to elucidate the precise mechanisms through which *FAIM2* interacts with obesity‐related factors and how these interactions may influence MS. Understanding these complex relationships could provide valuable insights into the development of targeted interventions aimed at mitigating the effects of obesity on MS progression.

### 3.4 *FTO* Gene Polymorphisms and MS

The *FTO* gene has attracted significant attention in MS research due to its established role in obesity as a recognized risk factor for MS (Czajkowski et al. 2020; Davis et al. 2014; C. Huang et al. 2023). The *FTO* gene is known to regulate energy homeostasis and adiposity and influence processes related to fat accumulation and BMI. The effects of *FTO* on obesity susceptibility may, in turn, impact the risk and progression of MS, highlighting its potential role in the interplay between metabolic disorders and neuroinflammatory conditions such as MS (Czajkowski et al. 2020; C. Huang et al. 2023). Given the established connection between obesity and both the onset and progression of MS, understanding the influence of *FTO* gene variants within this context is crucial.

Emerging evidence suggests that specific polymorphisms in the *FTO* gene may not only elevate the risk of obesity but also exacerbate the inflammatory pathways underlying MS pathology. *FTO* gene risk alleles may lead to more severe MS manifestations and outcomes (Al‐Serri et al. 2019; Panera et al. 2022; Parveen et al. 2022; Popović et al. 2023). Elucidating the interactions of *MS* and the *FTO* gene may provide new insights into the pathophysiology of MS and identify potential therapeutic targets.

In MS patients, the interaction between *FTO* gene variants and obesity‐related metabolic disturbances, such as insulin resistance and chronic low‐grade inflammation, may be pivotal in driving disease pathogenesis. Variants of the *FTO* gene could modulate key inflammatory pathways, thereby amplifying the autoimmune response characteristic of MS and potentially resulting in a more aggressive disease course. This complex relationship highlights the importance of developing personalized management strategies for patients with elevated BMI, aiming to optimize therapeutic outcomes (Chen et al. 2023; M. Li, Chi, et al. 2022; Parveen et al. 2022; Popović et al. 2023).

In the investigation conducted by Davis et al., the interplay between homocysteine levels and the *FTO* rs9939609 was explored in the context of MS, a condition previously linked to both homocysteine and obesity as significant risk factors. The study encompassed 114 MS patients alongside 195 matched controls, with a focused subgroup of 60 patients and 87 controls undergoing comprehensive analysis for vascular risk factors. Notably, the presence of the *FTO* rs9939609 A‐allele was significantly correlated with elevated homocysteine concentrations in MS patients, a relationship absent in the control group. Furthermore, homocysteine levels exhibited a positive correlation with BMI and total cholesterol. Dietary intake of folate emerged as a critical factor, with higher consumption linked to significant reductions in both homocysteine and BMI. The study also highlighted that increased BMI was associated with higher saturated/trans fat consumption and lower physical activity levels. Additionally, a diet rich in fruits and vegetables was shown to positively influence the expanded disability status scale scores, while smoking exacerbated MS‐associated disability. This research underscores the moderating impact of MS on the connection between *FTO* rs9939609 polymorphisms and homocysteine levels, emphasizing the gene's role in optimal health outcomes in MS patients (Davis et al. 2014).

Furthermore, exploring the *FTO* gene's role in MS opens potential therapeutic avenues. For example, targeting the metabolic and inflammatory pathways influenced by *FTO* could mitigate some of the adverse effects of obesity on MS progression. Lifestyle interventions such as weight management and anti‐inflammatory diets may be particularly beneficial for MS patients with high‐risk *FTO* genotypes. Future studies should aim to unravel the intricate interactions between *FTO*, obesity, and MS, paving the way for more effective prevention and treatment strategies (Flores‐Dorantes et al. 2020; Szalanczy et al. 2022; Xu et al. 2023).

### 3.5 *GNPDA2* Gene Polymorphisms and MS

GNPDA2 plays a critical role in carbohydrate metabolism through its enzymatic activity, catalyzing the deamination of glucosamine‐6‐phosphate (GlcN6P) to fructose‐6‐phosphate (Fru6P) and ammonia, functioning in the reverse direction of the rate‐limiting enzyme of the hexosamine biosynthesis pathway (HBP), glutamine‐fructose‐6‐phosphate aminotransferase (GFAT) (Wu et al. 2019). The HBP is a crucial nutrient‐sensing pathway involved in modulating cellular responses to metabolic changes, particularly under hyperglycemic conditions (Paneque et al. 2023; Wu et al. 2019). GWAS have underscored the importance of *GNPDA2* by linking it with obesity and type 2 diabetes (Kong et al. 2014). Despite these associations, the precise metabolic role of *GNPDA2*, particularly in the regulation of the CNS, remained insufficiently understood.

A study by Cappelletti et al. (2022) examined the role of CD4+ T cell activation in MS through a proteomic approach and observed that GNPDA2 is significantly downregulated in unstimulated CD4+ T cells from MS patients compared to healthy controls, suggesting that reduced *GNPDA2* expression may impact cellular processes, including glycosylation and energy production, which could contribute to immune dysregulation in MS. Another study investigating potential drugs for treating MS through transcriptomic analysis of immune cells identified key differentially expressed genes (DEGs) in MS patients, leading to the discovery of six candidate drugs, including fostamatinib and aspirin. Notably, *GNPDA2* was consistently differentially expressed in MS patients, supporting the findings of the study and reinforcing the potential of drug repositioning as a therapeutic strategy for MS (Yin et al. 2022).

Additionally, research has found that a genetically determined increase in BMI is associated with a 41% higher risk of MS, with *GNPDA2* SNPs identified as one of the BMI‐associated risk factors (Mokry et al. 2016). This suggests a potential causal link between obesity and MS development. However, although the *GNPDA2* was significantly associated with BMI in the GIANT consortium's data, its association with MS in the International MS Genetics Consortium (IMSGC) data was not statistically significant (OR: 1.02, 95% CI 0.99–1.05, *p* = 0.28), indicating that while *GNPDA2* is linked to BMI, its direct role in increasing MS risk may be limited (Mokry et al. 2016).

### 3.6 *MC4R* Gene Polymorphisms and MS

*MC4R* is a critical component of the CNS that plays a pivotal role in regulating energy balance and food intake (Seeley et al. 2004). *MC4R* is one of the five known melanocortin receptors (*MC1R–MC5R*) and is predominantly expressed throughout the CNS (Voisey et al. 2003). Beyond its metabolic functions, *MC4R* is implicated in anti‐inflammatory processes within the brain, although the precise mechanisms were initially unclear (Lasaga et al. 2008). Activation of *MC4R* by α‐melanocyte‐stimulating hormone (α‐MSH) and its analog NDP‐MSH has been shown to increase BDNF mRNA and protein levels in rat astrocytes. This effect is mediated through the cyclic AMP‐protein kinase A‐cAMP response element‐binding protein (cAMP‐PKA‐CREB) signaling pathway and can be inhibited by specific pathway blockers. Importantly, while both α‐MSH and inflammatory stimuli (LPS + IFN‐γ) activate CREB, only α‐MSH significantly enhances BDNF expression without engaging the NF‐κB pathway, highlighting a distinct anti‐inflammatory action (Caruso et al. 2012).

Melanocortin receptors, particularly *MC4R*, are vital for neuroprotection and inflammation regulation, making them highly relevant in the pathophysiology of MS. Activation of *MC4R* by adrenocorticotropic hormone (ACTH) and α‐MSH has demonstrated protective effects on oligodendrocytes against damage from oxidative stress and excitotoxicity, while simultaneously promoting neurogenesis and remyelination. These findings underscore the therapeutic potential of *MC4R* activation in MS (Lisak and Benjamins 2017). Additionally, *MC4R* activation modulates immune responses and neurotransmitter release, resulting in reduced immune cell activity and enhanced neuroprotection, which are crucial in managing MS (Arnason et al. 2013).

A study by Kamermans et al. further explored the therapeutic potential of *MC4R* in MS by targeting astrocytes, which are key players in disease progression and are found to have elevated *MC4R* expression in active MS lesions. The study demonstrated that activating *MC4R* with the selective agonist setmelanotide reduced the reactive, pro‐inflammatory state of astrocytes in vitro and significantly increased the production of anti‐inflammatory interleukins (IL‐6 and IL‐11), likely through enhanced CREB phosphorylation. Moreover, the medium from setmelanotide‐treated astrocytes was shown to influence macrophages, promoting an anti‐inflammatory phenotype (Kamermans et al. 2019).

### 3.7 *BDNF* Gene Polymorphisms and MS

*BDNF* is a crucial protein within the neurotrophin family, which includes nerve growth factor (NGF), neurotrophin‐3 (NT‐3), and NT‐4/5. *BDNF* levels in healthy individuals exhibit substantial variability, with mean plasma concentrations around 92.5 pg/mL. Notably, higher levels are observed in women, although these levels decline with age in both genders. *BDNF* is widely distributed across various brain regions and peripheral tissues, such as the gastrointestinal tract, lungs, heart, spleen, liver, and vascular smooth muscle cells, with higher concentrations found in the colon, bladder, and lungs compared to the skin and brain (Mobed et al. 2023). Neurotrophins regulate key aspects of nerve cell growth and function, including differentiation, survival, apoptosis, and synaptic plasticity (E. Huang and Reichardt 2001).

*BDNF* is produced by neurons, reactive astrocytes, and immune cells, such as T cells and macrophages, within MS lesions. Its receptor, TrkB, is upregulated near MS plaques, suggesting a role in neuronal protection and myelin integrity (Hohlfeld 2008; Nociti and Romozzi 2023). Immune cells within MS lesions, particularly those with anti‐inflammatory phenotypes, also secrete *BDNF*, contributing to myelin repair and reducing macrophage activity (Brück 2005). Some studies have proposed that chronic MS progression may influence *BDNF* levels (Kim 2023). However, most research has focused on RRMS, and it remains unclear whether *BDNF* plays a similar role in PPMS or if the inflammatory processes in PPMS differ significantly.

Glatiramer acetate (GA), a therapy for MS, may modulate *BDNF*'s neuroprotective effects. GA is believed to shift T‐cell responses from pro‐inflammatory Th1 to anti‐inflammatory Th2 phenotypes. GA‐specific T cells have been shown to produce *BDNF*, suggesting that GA could exert neuroprotective or neuroregenerative effects via localized *BDNF* release in MS lesions (Hohlfeld 2004). Additionally, research on fibroblast growth factor receptors (FGFRs) in oligodendrocytes during experimental autoimmune encephalomyelitis (EAE) suggests that FGFR1 and FGFR2 modulate *BDNF*/TrkB signaling, affecting inflammation and remyelination. Deletion of FGFR1 leads to increased *BDNF*/TrkB activity, promoting remyelination, while FGFR2 deficiency activates alternative pro‐myelinating pathways, reducing myelin degeneration (Rajendran et al. 2021).

Elevated levels of TNF‐α in the CNS are associated with MS disease activity and blood‐brain barrier (BBB) disruption. TNF‐α induces the expression of *BDNF* and NGF, both of which contribute to myelination and repair processes in the CNS. *BDNF* is known for its positive effects on myelination. NGF modulates TNF‐α activity by regulating its interactions with TNF receptors. At lower NGF levels, TNF‐α predominantly interacts with TNFR1, resulting in pro‐inflammatory and apoptotic effects. In contrast, higher NGF levels favor TNFR2 interaction, which supports myelin repair. NGF also promotes *BDNF* expression in the dorsal root ganglia (DRG) and CNS, further aiding myelin repair (Acosta et al. 2013). Interestingly, elevated cytokine levels in MS are linked to reduced circulating *BDNF* and with poorer cognitive outcomes, suggesting that *BDNF* is crucial for CNS myelination and could hold therapeutic potential in MS.

MicroRNAs (miRNAs) are also implicated in MS pathogenesis, with over 500 miRNAs reported as dysregulated in the disease. Specific miRNAs, such as miR‐155‐5p, miR‐125a, and miR‐191, have significant interactions with *BDNF*. For instance, a negative relationship between miR‐155‐5p and *BDNF* mRNA levels has been observed in MS animal models, indicating that reducing miR‐155‐5p could enhance *BDNF* expression. Similarly, miR‐125a and miR‐191 are inversely associated with *BDNF* expression, suggesting these miRNAs may regulate oligodendrocyte maturation and myelination (Eyileten et al. 2021).

*BDNF* plays a complex role in MS, affecting both neuroprotection and the persistence of autoreactive T cells. It is produced by neural tissues and immune cells, including CD4+ and CD8+ T cells, with levels rising during active inflammation, linking with MRI findings and clinical relapses. BDNF primarily exerts its effects through the TrkB receptor, especially the TrkB‐TK isoform, which is involved in neuroprotection and T‐cell survival. While *BDNF* supports neuronal survival and remyelination, it may also contribute to the persistence of autoreactive T cells by enhancing their resistance to activation‐induced cell death (AICD). This dual role presents a challenge, as BDNF facilitates neuroprotection but may also sustain deleterious immune responses in MS. The impact of immunomodulatory therapies, such as GA and interferon‐beta (IFN‐β), on *BDNF* production adds further complexity, with GA shown to elevate *BDNF* levels, potentially enhancing neuroprotection, whereas the effects of IFN‐β on *BDNF* remain unclear (De Santi et al. 2011).

Although *BDNF* has the potential to enhance remyelination, its benefits in MS are limited by the chronic inflammatory environment, and conventional immunosuppressive therapies may reduce its neuroprotective effects (Hohlfeld et al. 2000). Additionally, *BDNF* polymorphisms, such as Val66Met, influence MS progression and cognitive outcomes, with the Met allele unexpectedly association with improved cognitive function in MS (Schirò et al. 2022). Other factors, such as physical activity and gut microbiota, also influence *BDNF* levels, potentially enhancing neuroprotection and mitigating MS symptoms (Schirò et al. 2022).

*BDNF* is pivotal in promoting remyelination and repair processes, potentially slowing MS progression (KhorshidAhmad et al. 2016). It also plays a role in the neuroprotective and anti‐inflammatory effects seen in therapeutic approaches like neural stem cell transplantation (Y. Li, Li, et al. 2022). Despite its neuroprotective properties, the clinical application of *BDNF* is hindered by its short half‐life and difficulty crossing the BBB. To address these challenges, researchers are investigating the use of genetically engineered stem cells to deliver *BDNF* directly into the CNS. In MS models, this strategy has shown promise by delaying disease onset, reducing symptoms, and promoting remyelination, though further research is needed to confirm its efficacy in humans (Feng and Gao 2012).

The therapeutic potential of *BDNF* may be further enhanced when combined with other trophic factors, such as ciliary neurotrophic factor (CNTF) or fibroblast growth factor (FGF), and by modulating cytokine networks involved in the inflammatory responses characteristic of MS (Ebadi et al. 1997). Estrogen has demonstrated neuroprotective effects in animal models by shielding oligodendrocytes from cytotoxic damage, potentially upregulating *BDNF*. However, elevated estrogen levels, particularly with high estradiol and low progesterone, have been linked to increased MS disease activity, likely due to estrogen's interaction with other inflammatory mediators, such as mast cells. This suggests that while *BDNF* is generally neuroprotective, its modulation by estrogen in MS may yield both beneficial and adverse effects, contingent on the balance of other factors (Sohrabji and Lewis 2006). Furthermore, Fingolimod, an approved MS treatment, has exhibited neuroprotective effects by enhancing *BDNF* signaling, which is vital for neuronal survival, growth, and synaptic plasticity, critical components for memory and cognitive function (Leßmann et al. 2023).

### 3.8 *NPC1* Gene Polymorphisms and MS

*NPC* is an infrequent, autosomal recessive disorder marked by lipid storage anomalies, predominantly triggered by mutations in the *NPC1* or *NPC2* genes. These genetic alterations interfere with the normal trafficking processes within endosomes and lysosomes, resulting in the intracellular accumulation of cholesterol and other lipids. Clinically, *NPC* presents a diverse range of symptoms, from neonatal hepatic dysfunction to progressive neurodegenerative conditions in adults. The *NPC1* gene, in particular, has been the focus of extensive research due to its critical role in maintaining cellular lipid equilibrium and its significant impact on neurodegenerative mechanisms. Recent studies have also underscored the involvement of *NPC1* in pathways associated with obesity, illustrating that mutations in this gene may lead to metabolic imbalances and heightened vulnerability to neurodegenerative disease. Elucidating the links between *NPC1* mutations, obesity, and neurodegeneration could open new avenues for therapeutic interventions targeting these interrelated pathways (Tortelli et al. 2014; Vanier 2010; Zech et al. 2013). However, no studies have explored the role of *NPC1* gene polymorphisms in individuals with MS.

### 3.9 Future Research Directions

As we advance our understanding of the interplay between obesity‐associated genes and MS, several key areas warrant further investigation to address existing gaps and refine therapeutic strategies. Firstly, while current research highlights associations between specific genetic polymorphisms and MS risk, the underlying mechanisms linking these genes to disease pathology remain poorly defined. Future studies should focus on elucidating the molecular pathways through which obesity‐related genetic variants influence MS susceptibility and progression. This includes investigating how these SNPs influence the interaction of genes with environmental factors, such as diet and lifestyle, and how they affect inflammatory responses and neurodegeneration in MS. Employing advanced genomic techniques, such as *CRISPR‐Cas9* gene editing and high‐throughput sequencing, could provide deeper insights into these interactions and identify novel therapeutic targets.

Additionally, the clinical implications of obesity‐associated gene polymorphisms in MS require further exploration. While the current literature provides valuable evidence, there is a need for longitudinal studies that assess the impact of these genetic factors on the disease course and treatment outcomes over time. Research should also focus on integrating genetic data with clinical and biomarker profiles to develop personalized medicine approaches tailored to individuals’ genetic risk profiles. This approach could help in designing targeted interventions that address both obesity and MS, potentially improving patient outcomes. Furthermore, exploring the role of epigenetic modifications and gene–environment interactions in MS pathogenesis could reveal new avenues for therapeutic intervention. Expanding research to include diverse populations and considering the influence of socioeconomic factors on obesity and MS could also enhance the generalizability and applicability of findings. Ultimately, a multidisciplinary approach combining genomics, clinical research, and patient‐centered studies will be crucial in advancing our understanding and treatment of MS in the context of obesity.

## 4 Conclusion

In conclusion, this systematic literature review highlighted the critical roles that obesity‐associated genes play in the susceptibility and progression of MS. By examining key genetic factors such as *FTO*, *GNPDA2*, *MC4R*, and *BDNF* genes, we have illuminated their complex interactions with metabolic and inflammatory pathways that contribute to MS pathology. Our findings demonstrate that these genes not only influence obesity but also have a significant impact on MS risk and disease course, revealing novel insights into the genetic underpinnings of this condition. This comprehensive systematic review underscores the importance of understanding these genetic associations, offering valuable perspectives on how metabolic and inflammatory processes intertwine with neurodegenerative diseases. Ultimately, this study enhances our knowledge of MS pathogenesis and emphasizes the need for a more integrated approach to research, paving the way for future advancements in understanding and managing this challenging disease.

## Author Contributions

**Ali Jafari**: investigation, methodology, project administration, visualization, writing – original draft, writing – review and editing, validation. **Sara Khoshdooz**: writing – original draft, data curation, investigation. **Melika Arab Bafrani**: writing – original draft, data curation, investigation. **Farnush Bakhshimoghaddam**: writing – original draft, data curation. **Hamid Abbasi**: conceptualization, methodology, investigation, project administration, visualization, supervision, writing – original draft, writing – review and editing, validation, resources. **Saeid Doaei**: conceptualization, visualization, supervision, writing – review and editing, resources.

## Conflicts of Interest

The authors declare no conflicts of interest.

### Peer Review

The peer review history for this article is available at https://publons.com/publon/10.1002/brb3.70439.

## Supporting information



Supporting Information

Supporting Information

## Data Availability

All relevant data are provided within the manuscript and supporting information.
