# Supplementary material for: Uncovering the Causal Link Between Obesity‐Associated Genes and Multiple Sclerosis: A Systematic Literature Review
Source: Brain Behav. 2025 Apr 7;15(4):e70439. doi: 10.1002/brb3.70439 (PMC11975544; doi:10.1002/brb3.70439)
Supplement: Supplementary file 1 — Supporting Information [file BRB3-15-e70439-s002.docx]

**Uncovering the Causal Link between Obesity-associated Genes and Multiple Sclerosis: A Literature Review**

**Contents**

[**Table S1.** Search strategy to find potential eligible studies (July 2024) 2](#_Toc171962312)

| **Table S1.** Search strategy to find potential eligible studies (July 2024) |
| --- |

**PubMed = 406**

Limits: -

|  | **Descriptors** |
| --- | --- |
| #1 | (((((((((((("fas apoptotic inhibitory molecule 2"[Title/Abstract]) OR ("FAIM2"[Title/Abstract])) OR ("Niemann-Pick disease type C1"[Title/Abstract])) OR ("NPC1"[Title/Abstract])) OR ("Fat mass and obesity-associated"[Title/Abstract])) OR ("FTO"[Title/Abstract])) OR ("melanocortin-4 receptor"[Title/Abstract])) OR ("MC4R"[Title/Abstract])) OR ("brain-derived neurotrophic factor"[Title/Abstract])) OR ("BDNF"[Title/Abstract])) OR ("glucosamine-6-phosphate deaminase 2"[Title/Abstract])) OR ("GNPDA2"[Title/Abstract])) AND ("multiple sclerosis"[Title/Abstract]) |

**Web of Science = 663**

Limits: -

|  | **Descriptors** |
| --- | --- |
| #1 | (((((((((((TS=("fas apoptotic inhibitory molecule 2")) OR TS=("FAIM2")) OR TS=("Niemann-Pick disease type C1")) OR TS=("NPC1")) OR TS=("Fat mass and obesity-associated")) OR TS=("FTO")) OR TS=("melanocortin-4 receptor")) OR TS=("MC4R")) OR TS=("brain-derived neurotrophic factor")) OR TS=("BDNF")) OR TS=("glucosamine-6-phosphate deaminase 2")) OR TS=("GNPDA2") AND TS=("multiple sclerosis") |

**Scopus = 1179**

Limits: -

|  | **Descriptors** |
| --- | --- |
| #1 | ( TITLE-ABS-KEY ( "fas apoptotic inhibitory molecule 2" ) OR TITLE-ABS-KEY ( "FAIM2" ) OR TITLE-ABS-KEY ( "Niemann-Pick disease type C1" ) OR TITLE-ABS-KEY ( "NPC1" ) OR TITLE-ABS-KEY ( "Fat mass and obesity-associated" ) OR TITLE-ABS-KEY ( "FTO" ) OR TITLE-ABS-KEY ( "melanocortin-4 receptor" ) OR TITLE-ABS-KEY ( "MC4R" ) OR TITLE-ABS-KEY ( "brain-derived neurotrophic factor" ) OR TITLE-ABS-KEY ( "BDNF" ) OR TITLE-ABS-KEY ( "glucosamine-6-phosphate deaminase 2" ) OR TITLE-ABS-KEY ( "GNPDA2" ) ) AND TITLE-ABS-KEY ( "multiple sclerosis" ) |

**Embase = 603**

Limits: -

|  | **Descriptors** |
| --- | --- |
| #1 | ('fas apoptotic inhibitory molecule 2':ti,ab OR 'faim2':ti,ab OR 'niemann-pick disease type c1':ti,ab OR 'npc1':ti,ab OR 'fat mass and obesity-associated':ti,ab OR 'fto':ti,ab OR 'melanocortin-4 receptor':ti,ab OR 'mc4r':ti,ab OR 'brain-derived neurotrophic factor':ti,ab OR 'bdnf':ti,ab OR 'glucosamine-6-phosphate deaminase 2':ti,ab OR 'gnpda2':ti,ab) AND 'multiple sclerosis':ti,ab |

**Cochrane = 67**

Limits: -

|  | **Descriptors** |
| --- | --- |
| #1 | ("fas apoptotic inhibitory molecule 2" OR "FAIM2" OR "Niemann-Pick disease type C1" OR "NPC1" OR "Fat mass and obesity-associated" OR "FTO" OR "melanocortin-4 receptor" OR "MC4R" OR "brain-derived neurotrophic factor" OR "BDNF" OR "glucosamine-6-phosphate deaminase 2" OR "GNPDA2"):ti,ab,kw AND "multiple sclerosis":ti,ab,kw |
