# Supplementary material for: Uncovering the Causal Link Between Obesity‐Associated Genes and Multiple Sclerosis: A Systematic Literature Review
Source: Brain Behav. 2025 Apr 7;15(4):e70439. doi: 10.1002/brb3.70439 (PMC11975544; doi:10.1002/brb3.70439)
Supplement: Supplementary file 2 — Supporting Information [file BRB3-15-e70439-s001.docx]

**Table S2.** Risk of bias assessment of the selected studies based on NOS tool.

| **Study** | **Risk of bias items** | | | **Overall** |  |
| --- | --- | --- | --- | --- | --- |
|  | **Selection^a^** | **Comparability^b^** | **Exposure/Outcome^c^** |  | **Quality classification** |
| Al-Serri et al. 2019 | *** | * | *** | 7 | High |
| Kamermans et al. 2019 | **** | ** | *** | 9 | High |
| Davis et al. 2014 | *** | * | *** | 7 | High |

^a^ Selection is counted from 0 to 4 stars according to the sufficient case/cohort definition, case/cohort representativeness, ascertainment and selection of non-exposed or exposed cases.

^b^ Comparability is counted from 0 to 2 stars according to the design or analysis of comparability of cases and controls.

^c^ Exposure/outcome is counted from 0 to 3 stars based on outcome assessment and exposure ascertainment

**Table S3.** Risk of bias assessment of the selected MR study based on STROBE-MR tool.

| Mokry et al. 2016 | **Domain** | **Relevance^1^** | **Independence^2^** | **Exculsion-Restriction^3^** | **Pleiotropy^4^** | **Population stratification^5^** | **Data quality^6^** | **Overall quality** | **Overall score (quality)** |
| --- | --- | --- | --- | --- | --- | --- | --- | --- | --- |
|  | **Assessment** | Fully met | Fully met | Fully met | Fully met | Fully met | Fully met | Fully met | 7/7 (High) |
|  | **Score** | 1 | 1 | 1 | 1 | 1 | 1 | 1 |  |

^1^ Evaluation of single nucleotide polymorphisms (SNP) selection criteria and statistical significance.

^2^ Assessment of genetic association with confounders and control for population stratification.

^3^ Use of sensitivity analyses and pleiotropy assessment.

^4^ Evaluation of potential pleiotropic effects using MR-Egger regression and weighted median methods.

^5^ Control for population stratification and geographical correlations.

^6^ Validation of SNPs and assessment of proxy SNPs for accuracy.
